# Meals That Differ in Nutrient Composition and Inflammatory Potential Do Not Differentially Impact Postprandial Circulating Cytokines in Older Adults above a Healthy Weight

**DOI:** 10.3390/nu14071470

**Published:** 2022-04-01

**Authors:** Stephanie Cowan, Simone Gibson, Andrew J. Sinclair, Helen Truby, Aimee L. Dordevic

**Affiliations:** 1 Department of Nutrition, Dietetics and Food, Monash University, Notting Hill 3168, Australia; stephanie.cowan@monash.edu (S.C.); simone.gibson@monash.edu (S.G.); andrew.sinclair@monash.edu (A.J.S.); 2 School of Human Movement and Nutrition Sciences, University of Queensland, Brisbane 4072, Australia; h.truby@uq.edu.au

**Keywords:** inflammation, cytokines, obesity, cardiovascular disease, postprandial, dietary inflammatory index

## Abstract

In this exploratory study, mixed meals specifically formulated to differ in inflammatory potential were tested to determine whether they could differentially impact circulating levels of inflammatory markers in adults above a healthy weight. Complete data were analyzed from 11 adults (6 males and 5 females) aged 54–63 years with median BMI of 30.0 (27.1–31.6) kg/m². In a crossover study design, each participant consumed an isocaloric (2.2 MJ) meal with either a low (Anti-meal), moderate (Neutr-meal), or high (Pro-meal) inflammatory potential. Fasting and postprandial blood samples were analyzed for plasma levels of IL-6, IL-1β, TNF-α, IL-10, and metabolic makers. Postprandial plasma IL-6, IL-1β, TNF-α, and IL-10 incremental areas under the curve (iAUC) were not different between the three meals (*p* > 0.05). There was a trend of an increase in IL-6 with time in all three meals, but no changes were obvious for the other measured cytokines. The Pro-meal induced an increased postprandial iAUC for triglycerides compared to the Anti-meal and Neutr-meal (*p* = 0.004 and *p* = 0.012, respectively). Single meals, regardless of their theoretical inflammatory potential, did not substantially shift circulating inflammatory markers, suggesting that longer-term dietary patterns are important rather than single dietary exposures in the pathology of metabolic conditions.

## 1. Introduction

Subclinical chronic inflammation is the underlying pathology of obesity-related chronic diseases, including cardiovascular diseases (CVD) and type 2 diabetes mellitus (T2DM), which continue to be the leading causes of morbidity and mortality worldwide [1,2]. Inflammation underpins natural decline in health with ageing [3,4,5], but increased adiposity is also a key driver of inflammation, and an unhealthy diet is a major modifiable risk factor that plays a central role in initiating the inflammatory cascade observed in obesity [6,7]. While the assessment of dietary patterns is essential for understanding the role of food in the pathogenesis of chronic disease, the acute postprandial response is reflective of metabolic health and can help us further understand the interplay between nutrients and tissues [8]. Consumption of meals that reduce postprandial inflammation ultimately decrease overall inflammatory status and protect against chronic metabolic disease. This is particularly important when investigating the inflammatory effects of food, with research indicating a need to better understand how nutritional composition of meals can acutely affect inflammatory markers independent of changes in adiposity [9].

Postprandial inflammation is partly mediated by insulin [10] and lipids [11], and this stress response is usually modest, with homeostasis being rapidly restored. However, in metabolically compromised individuals, such as older people and those who are well above a healthy weight, the postprandial inflammatory response may be further exaggerated by prolonged hyperinsulinemia, hyperglycemia [12], and hyperlipidemia [13]. Repeated consumption of meals that lead to adverse metabolic responses, chiefly those high in saturated fat and added sugars, may contribute to subclinical chronic inflammation in the long term [14,15].

In the state of subclinical chronic inflammation, fasting plasma levels of proinflammatory cytokines, such as tumor necrosis factor-α (TNF-α) and interleukins (IL-1β, IL-6), are increased, whereas anti-inflammatory cytokines, such as interleukin 10 (IL-10), are decreased [8]. This inflammation leads to the inhibition of tissue insulin signaling, resulting in dysregulated whole-body metabolism and elevated circulating levels of glucose and lipids [16,17,18]. Thus, these classical markers of inflammation are routinely measured to assess metabolic–inflammatory changes in postprandial meal studies. A number of these cytokines have been shown to acutely respond (≥2 h and ≤8 h) to a meal stimulus, albeit inconsistently [19]. Interleukin-6 is the only classical inflammatory marker known to consistently increase with consumption of meals that induce metabolic stress (e.g., high-energy, high-fat, and high-carbohydrate foods) [19,20,21]. Regardless, these markers are the most frequently assessed inflammatory markers in postprandial studies to date [8].

The postprandial proinflammatory effects of food have been demonstrated after a single high-fat meal [19,20], and while some studies have shown that the addition of specific food compounds (such as dietary antioxidants and polyunsaturated fats) can blunt this acute inflammatory response, the extent of this response has been inconsistent [22,23,24,25,26]. These inconsistences may be explained by the inability of existing research to account for the complex nutritional matrix that exists at meal times. Few studies have examined the postprandial inflammatory response of mixed meals with varying nutritional compositions on circulating cytokines. One study compared an American Heart Association (AHA) meal with a fast food style (FFS) meal [27]. The authors found there was a significant attenuation of circulating IL-1β concentration following the AHA meal compared to the FFS meal, but no differences were observed in levels of IL-6 or TNFα between the two meals [27]. Milan et al. [28] compared postprandial IL-6, IL-1β, and TNF-α after a high-fat McDonald’s restaurant meal and a low-fat Australian Guide to Healthy Eating meal in young (20–25 years) and older (60–75 years) adults who were healthy. The authors reported differences in inflammatory responses between age groups but not between meal types. It was reported that older healthy adults had increased levels of monocyte chemoattractant protein (MCP)-1 and IL-6 levels compared to younger adults. There is a paucity of studies that assess acute postprandial inflammatory effects of mixed meals that differ by more than the addition or removal of a single food or nutrient.

We aimed to determine whether mixed meals designed to vary in nutrient composition and inflammatory potential were capable of differentially impacting circulating levels of IL-6, IL-1β, TNF-α, and IL-10 in men and women at an increased risk of chronic metabolic disease, namely older adults above a healthy weight. The test meals were purposefully designed to be (a) an anti-inflammatory meal, (b) a typical healthy meal (neutral meal), or (c) a highly processed meal (proinflammatory meal). These three meals were assessed for inflammatory potential using the dietary inflammatory index (DII), which is a scoring tool that categorizes an individual’s diet on a continuum from maximally anti-inflammatory (score of −8.87) to maximally proinflammatory (score of +7.98) [29]. To the research team’s knowledge, this is the first time the DII has been used to determine the inflammatory potential of a single meal. We hypothesized that circulating inflammatory markers would be attenuated during the postprandial period following consumption of the anti-inflammatory meal compared to the neutral and proinflammatory meal.

## 2. Materials and Methods

### 2.1. Study Participants

The study was conducted at the Be Active Sleep Eat (BASE) facility at Monash University, Australia, between November 2017 and October 2018. Participants were recruited from the Melbourne metropolitan area via flyers and social media. Interested participants completed an online screening questionnaire (Qualtrics^®^ 2019, Provo, UT, USA) for assessment of inclusion and exclusion criteria. Eligible participants were men aged ≥50 and ≤75 years or postmenopausal women with a body mass index (BMI) > 25 kg/m^2^. Exclusion criteria were diagnosis of a chronic disease (such as CVD or T2DM); known allergies/intolerances to the study foods; loss of >10% total body weight in the 6 months preceding the study; smokers; history of drug or alcohol addiction; and use of anti-inflammatory medications, such as aspirin, steroids, or nonsteroidal anti-inflammatory drugs. Eligible participants were invited to the BASE facility to confirm eligibility with measurements including height (Holtain stadiometer to 0.1 cm, Holtain Ltd., Crosswell, Pembrokeshire, UK), weight (Seca scale 720 to 0.01 kg, Seca Group, Hamburg, Germany), and umbilical waist circumference (Figure Finder stretch resistant tape, Novel Products, Rockton, IL, USA). All measurements were taken in duplicate following standardized procedures and recorded to the nearest 0.1 decimal place. Blood pressure was measured using a digital blood pressure monitor (Welch Allyn ProBP 3400) on two occasions, a minimum of 5 min apart, with the participant sitting. This study was conducted in accordance with the Declaration of Helsinki. Ethics approval was granted through Monash University Human Research Ethics Committee (MUHREC no. 8773). All participants provided written, informed consent prior to commencement in the study.

### 2.2. Study Design and Procedures

**Figure 1:**
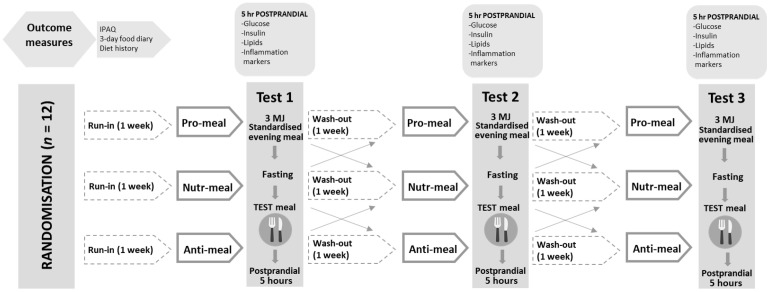
Crossover study design. IPAQ: International Physical Activity Questionnaire.

This pilot trial was retrospectively registered with the Australian New Zealand Clinical Trial Registry (ACTRN12620000525987, http://www.anzctr.org.au/) as a randomized, controlled, crossover design. There were a total of three study days, each separated by a one-week washout period (Figure 1). Participants were asked to abstain from alcohol consumption and avoid vigorous exercise for 24 h prior to the study days. They were also asked to consume a ready-made standardized meal that provided 3 MJ of energy (carbohydrates total energy (E) 55%, protein E 30%, and fat E 20%) the night prior to each study day. The standard meal comprised a Healthy Choice ravioli, a 50 g cheese and cracker pack, and 200 mL of apple juice. After fasting for 12 h overnight (water was permitted ad libitum), participants arrived at the test center by 9:00 a.m. Prior to collecting baseline measures, participants were asked if they had experienced any cold- or flu-like symptoms in the past seven days, and study days were rescheduled if required. Anthropometric measurements were taken, and the study nurse then inserted an indwelling catheter into the antecubital vein. Blood draws were obtained at baseline (fasting), and breakfast was provided to participants, which included one of three experimental meals: a proinflammatory meal (Pro-meal), a neutral meal (Neutr-meal), or an anti-inflammatory meal (Anti-meal). Participants were supervised to ensure all of the meal was consumed within 15 min. Participants were not permitted to consume any food or drink except water (provided ad libitum) during the study session. After participants had consumed the meal, blood was collected at 15, 30, 45, 60, 120, 180, 240, and 300 min. Plasma samples (for glucose, insulin, and inflammatory markers) were collected in EDTA tubes (BD, Melbourne, Australia) and immediately centrifuged at 1.5 RCF for 15 min at 4 °C. Serum samples (for lipids) were collected in SST-II tubes (BD, Melbourne, Australia) and allowed to clot at room temperature (30–60 min), then centrifuged at 1.3 RCF for 10 min at 22 °C. Plasma and serum samples were aliquoted and frozen at −80 °C until analysis.

### 2.3. Meal Development and Composition

The Pro-meal (a cheese and bacon croissant) was a highly processed meal obtained from commercially available products. The Neutr-meal (a chicken, vegetable, red kidney bean, and wholegrain pasta minestrone soup) was a typical healthy meal. The Anti-meal (a spiced chicken and red kidney bean curry served with brown rice, flaxseeds, and citrus kefir yoghurt) was an adaptation of the Neutr-meal specifically designed to optimize foods that contain anti-inflammatory compounds, including, but not limited to, β-carotene, eugenol, fiber, omega-3 fatty acids, vitamin A, vitamin E, and flavonoids.

All three meals were matched for energy content using FoodWorks nutrition software (FoodWorks 2017, Xyris Software, Queensland, Australia). Each of the three meals provided approximately 25% of an adult’s average daily energy intake (Table 1). The test meals were assessed for inflammatory status using the DII [29], a tool routinely used in research that categorizes an individual’s diet on a continuum from maximally anti-inflammatory (score of −8.87) to maximally proinflammatory (score of +7.98). In this study, it was used to create an inflammatory score for each test meal, with the results showing a score of +9.36 for the Pro-meal, −2.76 for the Neutr-meal, and −6.24 for the Anti-meal (Figure S1, Tables S1–S3). To the research team’s knowledge, this is the first time the DII has been used to determine the inflammatory potential of a single meal (Figure S1). It should be noted that the DII score generated for the Pro-meal sat outside of the theoretical bounds of −8.87 to +7.98. However, this was a novel application, and the purpose of generating DII scores for each meal was purely to emphasize their differences in inflammatory potential.

### 2.4. Baseline Nutritional Intake and Physical Activity Levels

Prior to their first study day, participants completed a validated physical activity questionnaire (International Physical Activity Questionnaire or IPAQ) [27]. The IPAQ comprises four items, namely leisure time, domestic and gardening activities, work-related activities, and transport-related activities. Total metabolic equivalent (MET)-minutes per week were calculated by summing the total MET-min per week for walking, moderate-intensity activity, and vigorous-intensity activity according to Guidelines for Data Processing and Analysis of the IPAQ [28].

Self-reported food records were obtained for three days prior to the participants’ first study day. A registered accredited practicing dietitian trained participants to weigh their food and/or estimate serving sizes using common household measures (e.g., cups and tablespoons) as well as record brand names and cooking methods. A detailed diet history was also recorded by the study dietitian encompassing important dietary pattern data, such as seasonal influences, frequency, and diversity of foods consumed. Collectively, the food records and diet histories were analyzed by FoodWorks nutrition software (FoodWorks 2017, Xyris Software, Spring Hill, Qld, Australia).

### 2.5. Anthropometry, Body Composition, and Blood Pressure

During the first testing visit, participants’ body composition was determined by a total body scan on the GE LUNAR iDXA narrow-angle dual-energy X-ray densitometer with SmartFAN™ (GE Medical, Software Lunar DPX enCORE 2012 version 14.0, Madison, WI, USA).

At each visit, participants’ height, weight, umbilical waist circumference, and blood pressure were measured as per screening procedures. Anthropometric measures were monitored to ensure weight stability throughout the study period.

### 2.6. Inflammatory and Metabolic Markers

The plasma inflammatory markers IL-6, IL-1β, TNF-α, and IL-10 were measured on site with MILLIPLEX^®^ map assays (MILLIPLEX MAP Human High Sensitivity T-Cell HSTCMAG-28SK; Merck Millipore, Billerica, MA, USA) using MAGPIX^®^ with xPONENT^®^ software (Luminex Corporation, Texas, United States) according to the manufacturer’s instructions. All samples were run in duplicate; if the duplicate values varied by >20%, the sample was reanalyzed. The intra-assay coefficient of variation (CV) was <10% for IL-6, TNF-α, IL-1β, and IL-10. In-house controls were used across all plates to standardize results between assays. Plasma CRP was analyzed at a hospital-certified pathology laboratory (Monash Medical Centre, Clayton, Australia) according to standard commercial enzyme-linked immunosorbent assay (ELISA) techniques.

Serum lipid concentrations were measured in house using Thermo Fisher Indiko clinical chemistry analyzer (Thermo Fisher Scientific, Vantaa, Finland) by enzymatic colorimetric methods using commercially available kits as per Thermo Fisher Scientific instructions (total cholesterol, 981813; triglycerides, 981786; LDL cholesterol, 981656; HDL cholesterol, 981823). The multicalibrators sCal (981831) and HDL/LDL calibrator (981657) were used to calibrate the analyzer, together with the control serums Nortrol (981043), Abtrol (981044), Lipotrol (981653), and Lipotrol abnormal (981907). All samples were run in duplicate; any sample with a coefficient of variation >10% was rerun. Intra-assay coefficient of variation was <5% for all lipids. The between-assay coefficient of variation was <5%. Plasma insulin and glucose were measured using standard enzyme-linked immunosorbent assay (ELISA) techniques at a hospital-certified pathology laboratory.

### 2.7. Randomisation, Blinding, and Statistical Analysis

A total of 12 adults were recruited to participate in this pilot study. As this was a novel protocol, there were no similar studies using energy-matched mixed meals with such vastly different nutrient compositions upon which a priori power calculation could be performed. The sequence for allocation to the order of meal type was generated using an electronic random number generator and concealed in a password-protected folder. It was generated by a researcher who was not involved in eligibility assessment; A.D. assigned participants to the meal order. The researcher responsible for data collection (S.C.) knew in advance the meal type allocation for each study day. Participants were not blinded to meal order in this study because the nature of the intervention meals prevented blinding or masking.

Biochemical data are presented as incremental area under the curve (iAUC), the preferred method for describing the acute metabolic response to meals [29,30]. Inflammatory markers and lipids were calculated over a five-hour period (0 to 300 min), whereas the iAUC for glucose and insulin were assessed over a three-hour period (0 to 180 min). Incremental AUC was calculated using the trapezoid rule, which ignores the area beneath the baseline concentration.

Statistical analyses were performed using SPSS software package for Windows (version 24.0; IBM Corporation, New York, NY, USA). Due to the small sample size, all data were treated as nonparametric during statistical analysis and reported as median (IQR). Friedman test followed by pairwise comparisons using Wilcoxon signed-rank test were used to make comparisons between the three test meals for iAUC glucose, insulin, lipids, and inflammatory markers. Wilcoxon signed-rank test was used to compare peak concentrations with baseline concentrations for inflammatory markers. The Mann–Whitney *U* test was used to compare measures between male and female participants. Data were visualized using GraphPad Prism software program for Windows (version 8.0.0; GraphPad Software, San Diego, CA, USA).

Due to missing samples during the Anti-meal challenge owing to complications with cannulation, one participant was excluded from analysis. Statistical significance was considered at *p* < 0.05.

## 3. Results

### 3.1. Participant Enrolment and Characteristics at Baseline

**Figure 2:**
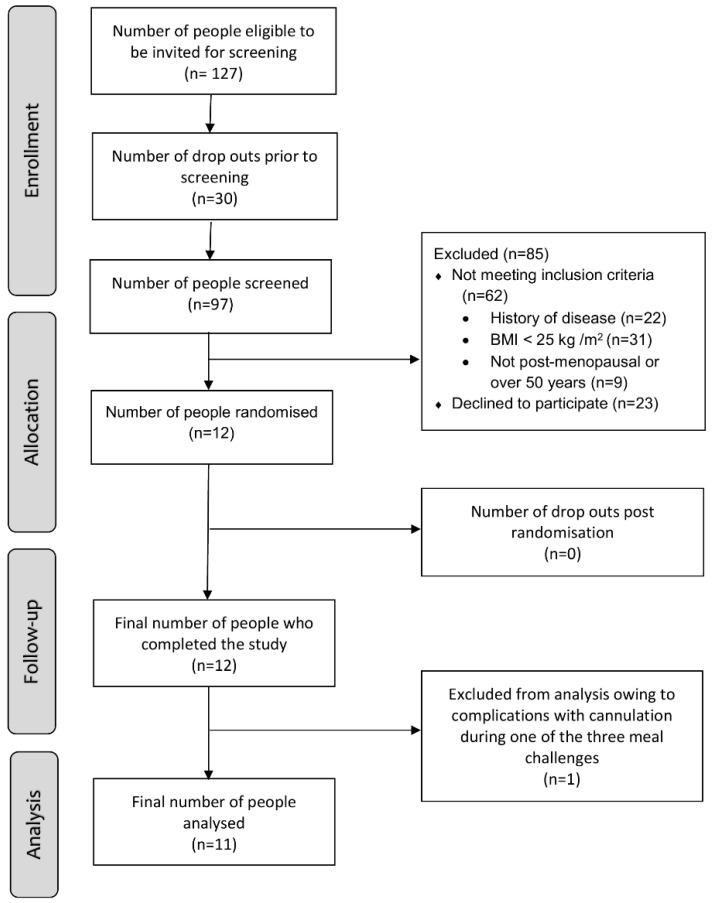
Participant flow through study.

A flowchart for recruitment is detailed in Figure 2. A total of 12 participants completed the study (*n* = 6 females, 6 males) with no attrition; however, one participant was removed from the final analysis, so complete data from the remaining 11 participants were analyzed. The median (IQR) age for the group (*n* = 11) was 61 (54–63) years and median BMI was 30.0 (27.1–31.6) kg/m² (Table 2). All 11 participants were White. There were no changes in the participants’ BMI (kg/m^2^) (Anti-meal 30.0 (27.1–31.6) vs. Neutr-meal 30.1 (27.1–31.5) vs. Pro-meal 29.7 (27.1–31.6), *p* = 0.739) or waist circumference (cm) (Anti-meal 101.0 (94.5–104) vs. Neutr-meal 101.1 (94.0–105.0) vs. Pro-meal 101.5 (93.9–104.5), *p* = 0.081) during the study period.

Females had a significantly higher proportion of total body fat (*p* = 0.028) and higher levels of fasting IL-6 (*p* = 0.008), IL-1β (*p* = 0.019), TNF-α (*p* = 0.014), total cholesterol (*p* = 0.023), and HDL cholesterol (*p* = 0.004) compared to males. Males had significantly higher levels of fasting glucose compared to females (*p* = 0.014). There were no significant differences in total cholesterol/HDL ratio or total visceral fat (g) between sexes.

Baseline dietary analyses revealed that participants consumed low intake of fruits (<1 serve/day) and vegetables (<4 serves/day) and high intake of refined grains (>50% of total grain intake) and saturated fat (13% total energy/day). The percentage energy intake from total fat was significantly higher for females compared to males (*p* = 0.045), although there were no significant differences observed between sexes for the other measured dietary parameters. Overall, participants had a high level of physical activity (>2000 MET-min/week) (Table 3).

### 3.2. Plasma Inflammatory Markers

**Figure 3:**
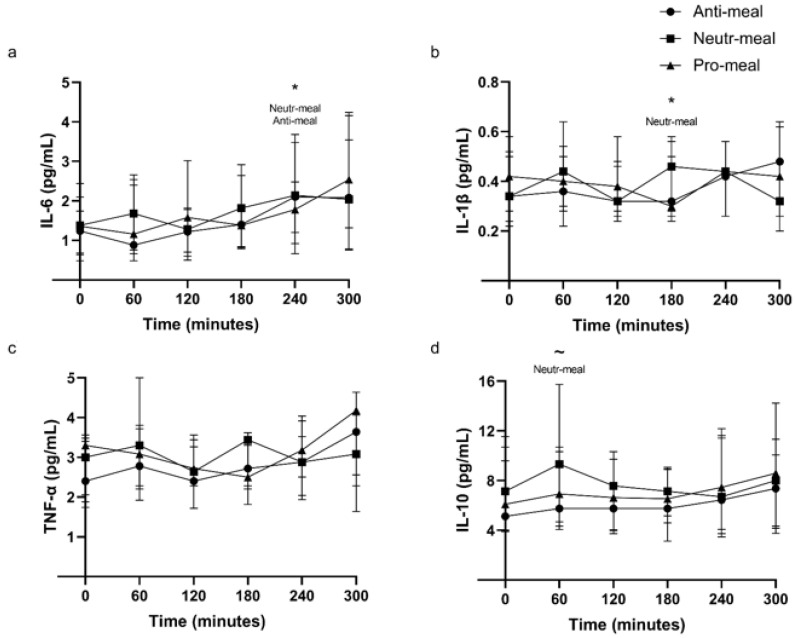
Postprandial plasma (**a**) IL-6, (**b**) Il-1β, (**c**) TNF-α, and (**d**) IL-10 concentrations after ingestion of Anti-meal, Neutr-meal, or Pro-meal. Data are presented as median ± IQR (*n* = 11), Friedman test followed by pairwise comparisons using Wilcoxon signed-rank test comparing iAUC between the test meals. * *p* < 0.05 and ~ *p* = 0.05 by Wilcoxon signed-rank test comparing peak with baseline concentrations within test meals. Abbreviations: Anti, anti-inflammatory; Neutr, neutral; Pro, proinflammatory.

Postprandial curves for plasma inflammatory markers are shown in Figure 3, and iAUC values are reported in Table 4. Fasting plasma IL-6, IL-1β, TNF-α, and IL-10 concentrations were not significantly different between the three meal challenges (*p* > 0.05 for all) (Figure 3).

There were no significant postprandial differences in iAUC for IL-6, IL-1β, IL-10, and TNF-α between the three test meals (Table 4). The peak IL-6 concentrations at 240 min were increased compared to fasting levels after the Neutr-meal (1.38 (0.48–2.1) vs. 2.14 (0.92–3.48), *p* = 0.017) and Anti-meal (1.24 (0.68–2.44) vs. 2.10 (0.66–3.68), *p* = 0.016). The peak IL-1β concentration at 180 min was increased compared to fasting levels after the Neutr-meal (0.34 (0.28–0.52) vs. 0.46 (0.28–0.56), *p* = 0.011). The peak IL-10 concentration at 60 min was increased compared to fasting levels after the Neutr-meal (6.04 (3.96–9.60) vs. 8.02 (4.06–15.74), *p* = 0.050).

There was a high degree of variability both within and between participants for all measured inflammatory makers (Figure S2). There were no significant differences between females and males in their postprandial response to the three test meals for all measured inflammatory markers (*p* ≥ 0.05 for all) (Table S4).

### 3.3. Plasma Glucose, Insulin, and Serum Lipids

**Figure 4:**
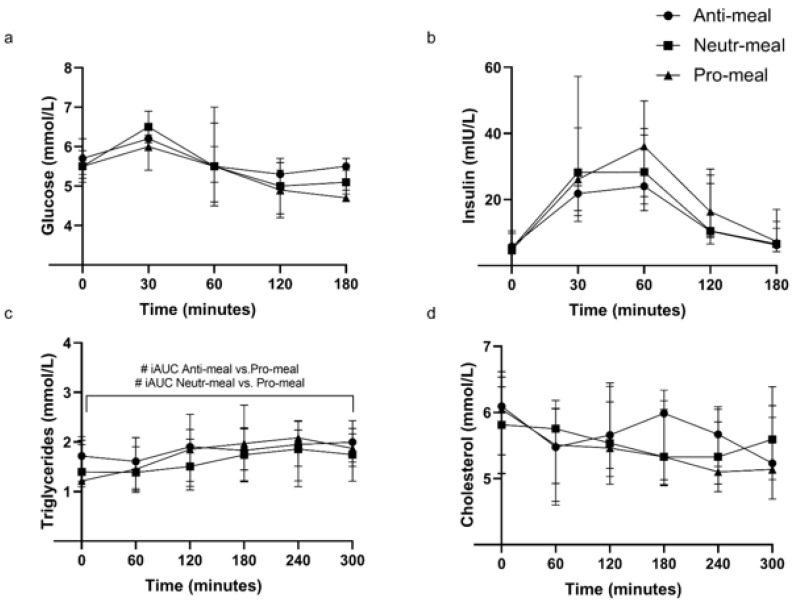
Postprandial (**a**) plasma glucose, (**b**) plasma insulin, (**c**) serum triglycerides, and (**d**) serum total cholesterol concentrations after ingestion of Anti-meal, Neutr-meal or Pro-meal. Data are presented as median ± IQR (*n* = 11). # *p* < 0.05 by Friedman test followed by pairwise comparisons using Wilcoxon signed-rank test comparing iAUC between test meals. Abbreviations: Anti, anti-inflammatory; Neutr, neutral; Pro, proinflammatory.

Postprandial curves for plasma glucose, insulin, and serum lipids are illustrated in Figure 4, and iAUC values are reported in Table 5.

Fasting glucose, insulin, total cholesterol, and triglyceride concentrations were not different between the three meal challenges (*p* ≥ 0.05 for all) (Figure 4).

The iAUC postprandial response for triglycerides was significantly different between the three test meals, with the Anti-meal and Neutr-meal producing a lower response than the Pro-meal (*p* = 0.004 and *p* = 0.012, respectively). There were no postprandial differences in iAUC for glucose, insulin, or total cholesterol (*p* ≥ 0.05 for all) (Table 5).

Postprandial iAUC responses to the three test meals for glucose, insulin, total cholesterol, and triglycerides were not different between females and males (*p* ≥ 0.05 for all) (Table S4).

## 4. Discussion

Despite the clear differences in nutrient composition and inflammatory potential of the meals, there were no differences in the postprandial responses of the circulating cytokines between the meals in older adults above a healthy weight. While glucose and insulin curves were similar between the meals, the proinflammatory meal elicited an increased triglyceride response in the participants, which is thought to be related to a postprandial inflammatory response [11]. However, this study showed that increased circulating triglycerides and differences in total amount and type of fat in the meals did not differentially impact the classical markers of inflammation, even in participants who were more likely to exhibit exaggerated postprandial cytokine responses due to their age and weight status [7,30].

The characterization of inflammatory response to meal composition that accounts for the whole food matrix and is not only focused on individual nutrients, such as fat [31], has increasing relevance for nutrition practice in older adults with obesity and increased risk of CVD and T2DM [9]. While previous research has largely focused on the effects of isolated nutrients, little is known about how the food matrix affects postprandial inflammation. For each nutrient, the bioaccessibility (fraction released during digestion), bioavailability (fraction absorbed), bioconversion (fraction converted to its active form), and bioactivity (actions within the body) are directly related to the food matrix [32]. It is well established that postprandial changes in blood glucose, and therefore insulin, depend on many factors, including interactions with fiber and polyphenols [33]. The addition of fiber to a meal containing carbohydrates can delay gastric emptying, thereby reducing the rate of intestinal glucose absorption [34], while dietary flavonoids blunt postprandial blood glucose levels via enzyme and transporter inhibition [35]. As postprandial inflammation is partly mediated by insulin [10] and lipids [11], other food components that impact these factors will likely also affect any inflammatory response. The meals in the present study were vastly different in composition. While the Anti-meal and Neutr-meal had a higher total carbohydrate content than the Pro-meal (53.1, 42.8, and 25.2 g, respectively), they also contained higher levels of fiber (19, 15.5, and 1.96 g, respectively) and polyphenols. Interactions occurring within the whole food matrix may explain why the only metabolic marker that exhibited a difference during the postprandial period was triglyceride levels, which were increased with consumption of the higher fat Pro-meal (containing ≥ 17 g more than both the Anti-meal and Neutr-meal). Both prolonged hyperglycemia [12] and hyperlipidemia [13] are related to postprandial inflammation, and it is possible that differences in the total fat load alone were not enough to generate discernible inflammatory responses between the test meals in the present study.

As IL-6 is the only marker of inflammation consistently reported to increase after a high-fat meal [19,20], it is the most commonly measured cytokine in postprandial research. A review by Emerson et al. [19] reported that a single high-fat meal (≥30% fat, ≥500 kcal) significantly increased levels of IL-6 in 32 of the 45 included studies. In healthy adults with mean fasting CRP levels of 1.5 mg/L, IL-6 was reported to peak at 2.9 pg/mL six hours post high-fat meal ingestion, which was more than a 100% increase from baseline levels [19]. A recent large (*n* = 1002) study measured postprandial inflammation over two meals (breakfast and lunch) using IL-6. This study reported increased IL-6 after breakfast and then a further increase after lunch but showed no association of IL-6 levels with metabolic markers such as glucose levels [21]. In the current study, Anti-meal and Neutr-meal produced a significantly lowered triglyceride iAUC compared to Pro-meal, but there were no significant differences in IL-6 iAUC between the three test meals. Changes in IL-6 over time may be explained by the cannulation process rather than the metabolic response to food [36]. One crossover study (postprandial period = 8 h) using a HFM challenge (39% fat) reported that while circulating IL-6 concentrations increased by 3.49 pg/mL in participants who were cannulated, only minimal changes in IL-6 were observed (0.36 pg/mL) in participants who were not cannulated but had blood taken via a single-use needle (before and 8 h after the meal) (*p* = 0.013) [37]. Furthermore, IL-6 is known to follow a circadian rhythm, where it increases across the day after a nadir (trough) around breakfast time and then a zenith (peak) around dinner time [38,39]. The peaks and troughs can reportedly differ by a few hours between different study populations and are dependent on sleep patterns [39]; thus, IL-6 is not a reliable marker to represent an inflammatory response to food.

To our knowledge, only one other crossover study (*n* = 11) by Devaraj et al. [27] investigated postprandial (time = 8 h) effects on circulating IL-6, IL-1β, and TNF-α concentrations using energy-matched meals with different macro- and micronutrient contents, hence accounting for the complex nutritional matrix that exists at meal times. This study showed a significant increase in postprandial IL-6 concentrations following both an American Heart Association (AHA) meal (8.6% fat) and a fast food style (FFS) meal (50.1% fat), but there was no difference between meals [27]. This is consistent with our study, which showed that postprandial IL-6 concentrations increased irrespective of differences in total fat content and are likely influenced by other factors. Similarly, consistent with the findings from our study, Devaraj et al. showed no significant change in TNF-α concentrations over time or with dietary treatment. Devaraj et al. did, however, demonstrate a significant increase in IL-1β concentrations after the FFS meal but not the AHA meal. While our study showed no difference in IL-1β response (iAUC) between the three test meals, comparison of peak concentrations with baseline showed a significant increase from baseline levels after the Neutr-meal but not the Anti-meal or Pro-meal. This unexpected response to the Neutr-meal suggests that further investigation of postprandial responses to mixed meals, rather than single nutrients such as fat, is needed.

It has been suggested that in healthy people, the compensatory anti-inflammatory effects of IL-10 can counteract proinflammatory cytokine responses [40]. Previous studies have reported that increased postprandial levels of IL-10 accompany increased proinflammatory cytokine responses to high-fat meals in healthy participants [41,42], indicating an attempt by the body to oppose meal-induced inflammation. In the present study, postprandial IL-10 levels did not differ between the meals, and conversely to previous literature, no increases were measured in participants following the high-fat Pro-meal. However, comparison of peak concentrations with baseline showed a borderline significant (*p* = 0.05) increase from baseline levels after the Neutr-meal, for which postprandial increases in IL-6 and IL-1β were also observed. Together, these data suggest that there may have been a differential cytokine response between the three test meals, but a larger sample size would be needed to confirm the results.

As this was a pilot study, it was likely not fully powered to detect significant changes in outcome measures (IL-6, TNF-α, IL-1β, and IL-10), which is a key limitation to the interpretation of these findings. However, the use of a crossover design meant that study participants were their own controls, which is a strength to determine whether the meals could differentially impact the circulation of inflammatory markers. We observed high variation in expression levels of the inflammatory markers, not only between but also within participants; thus, subclinical changes in response to nutrient flux would likely be difficult to detect in small sample sizes that are typical of postprandial studies.

It is well established that impaired metabolic flexibility, characterized by exaggerated responses in metabolic profiles following a meal challenge, is greater in individuals with increased metabolic dysregulation [43,44,45]. The participants in the present study were selected based on age and weight status to capture a group of individuals that were more likely to be at risk of chronic metabolic conditions. However, despite high levels of adiposity, a known mediator of inflammation [46,47], and generally unhealthy dietary patterns, the participants had high levels of physical activity and were relatively healthy based on other markers of chronic disease risk (including baseline inflammatory markers). The relatively healthy status of the participants may have impacted their inflammatory response to the meals.

Using circulating cytokines to investigate inflammatory changes to dietary stimulus may be more valuable in longer-term dietary intervention studies. Future research should consider markers of inflammation that are more sensitive and specific to nutrient flux, and it is likely that a panel of markers will be needed to capture the complexity of nutrient-driven stress responses. Mazidi et al. recently suggested glycoprotein acetylation (GlycA) as a suitable candidate [21]. Increased levels of GlycA have been associated with inflammation and chronic metabolic diseases [48,49,50], and Mazidi et al. showed that plasma levels increased during the postprandial period and were correlated with postprandial glucose and TG levels [21]. Markers of oxidative stress may also be useful candidates as they are intrinsically linked to inflammation and chronic disease [51] and transiently increase following energy-dense meals [52,53,54], likely due to macronutrient metabolism [55]. Other measures might include high-throughput transcriptomics, proteomics, and metabolomics, which are more sensitive in detecting subtle phenotypic changes [56,57].

## 5. Conclusions

We did not find evidence that consumption of single meals varying in nutrient composition and inflammatory potential differentially impact postprandial plasma levels of IL-6, IL-1β, TNF-α, or IL-10 in older adults above a healthy weight. This research further supports the need to look beyond the inflammatory effects of single nutrients, in particular fat, and emphasizes the importance of accounting for the whole food matrix when characterizing the postprandial inflammatory state. Examination of these classical inflammatory markers in postprandial studies may not be sufficient to design meals and diets that decrease overall inflammatory status to protect against chronic metabolic disease. To translate research into practical nutrition recommendations, future research should utilize mixed meals and employ sensitive measures of inflammatory status that capture the subtle phenotypic changes elicited at meal times.

## Figures and Tables

**Table 1:** Meal composition and dietary inflammatory index scores.

Nutrient	Pro-Meal ^1^	Neutr-Meal ^2^	Anti-Meal ^3^
Energy, kJ kcal	2280 545	2150 514	2335 558
% Fat, % kJ	64	25	35
% Carbohydrate, % kJ	19	33	36
% Protein, % kJ	17	34	19
Fat, % kJ	39.3	14.5	21.9
Saturated fat, g	20.4	6.2	3.4
Monounsaturated fat, g	13.7	5.3	9.4
Polyunsaturated fat, g	2.50	1.46	7.18
Carbohydrate, g	25.2	42.8	53.1
Fibre, g	1.96	15.5	19.0
Protein, g	22.2	43.1	25.7
DII score ^4^	+9.36	−2.76	−6.24

^1^ Cheese and bacon croissant. ^2^ Comprised mixed vegetables, red kidney beans, wholegrain pasta, and lean chicken breast cooked in a tomato-based stock and served with parmesan cheese. ^3^ Neutr-meal plus increased quantity of extra virgin olive oil, garlic, onion, and pepper; addition of turmeric, cumin, ginger, cloves, rosemary, thyme, saffron, silver beet, eggplant, tomato paste, and flaxseeds; and substitution of brown rice in place of wholemeal pasta and natural yoghurt and kefir in place of parmesan cheese. ^4^ The test meals were assessed for inflammatory status using the dietary inflammatory index (DII [29]), a tool routinely used in research that categorizes an individual’s diet on a continuum from maximally anti-inflammatory (score of −8.87) to maximally proinflammatory (score of +7.98). Abbreviations: Anti, anti-inflammatory; DII, dietary inflammatory index; Neutr, neutral; Pro, proinflammatory.

**Table 2:** Baseline physical and biochemical characteristics of study participants ^1^.

Variable	Total Population (*n* = 11)	Male (*n* = 5)	Female (*n* = 6)	Male vs. Female *p*-Value *^2^*
Waist circumference, cm	101 (95–104)	101 (97–110)	98 (90–107)	0.465
Waist-to-hip ratio	0.92 (0.88–0.97)	0.94 (0.89–1.00)	0.89 (0.86–0.97)	0.462
Total body fat, %	37 (30–43)	30 (27–36)	42 (36–47)	0.028
Total visceral fat, g	1285 (1120–2168)	1285 (1179–2425)	1553 (766–2387)	0.715
Systolic blood pressure, mm Hg	125 (120–137)	125 (122–140)	127 (117–132)	0.409
Diastolic blood pressure, mm Hg	78 (76–85)	77 (74–86)	80 (75–86)	0.855
Plasma glucose, mmol/L	5.50 (5.25–6.00)	5.70 (5.50–6.20)	5.35 (5.05–5.73)	0.014
Plasma insulin, mIU/L	5.30 (3.85–10.35)	5.30 (3.20–10.50)	5.50 (3.88–9.40)	0.885
Insulin resistance (HOMA-IR)	1.25 (0.94–2.73)	1.25 (0.85–3.20)	1.20 (0.97–2.40)	0.828
Serum TC, mmol/L	6.04 (5.19–6.56)	5.46 (5.08–6.17)	6.18 (5.80–6.95)	0.023
Serum LDL, mmol/L	4.02 (3.48–4.55)	3.69 (3.29–4.29)	4.15 (3.70–4.57)	0.065
Serum HDL, mmol/L	1.23 (1.05–1.76)	1.06 (0.96–1.26)	1.41 (1.19–1.84)	0.004
Serum TC/HDL ratio	4.65 (3.18–5.22)	4.65 (3.12–5.05)	4.46 (3.14–5.31)	0.800
Serum TAG, mmol/L	1.44 (1.18–2.03)	1.40 (1.21–1.95)	1.56 (1.13–2.20)	0.745
Plasma CRP, mg/L	1.00 (0.70–1.90)	0.70 (0.50–2.75)	1.05 (0.85–1.70)	0.313
Plasma IL-6, pg/mL	1.36 (0.64–1.92)	0.64 (0.44–1.24)	1.64 (1.32–2.58)	0.001
Plasma IL-1β, pg/mL	0.38 (0.25–0.51)	0.28 (0.18–0.44)	0.48 (0.34–0.54)	0.019
Plasma TNF-α, pg/mL	3.00 (1.96–3.48)	2.08 (1.74–3.00)	3.35 (2.76–3.52)	0.014
Plasma IL-10, pg/mL	6.04 (3.98–10.15)	4.90 (3.66–8.02)	6.88 (4.67–13.58)	0.051
Comorbidities, *n* (%) Hypertension Hypercholesterolemia	6 (55) 3 (27)	4 (67) 1 (17)	2 (33) 2 (33)	- -
Medication use, *n* (%) Antihypertensive Statins/fibrates Anticoagulants	6 (55) 3 (27) 2 (18)	4 (67) 1 (17) 1 (17)	2 (33) 2 (33) 1 (17)	- - -

^1^ Values are median (IQR) unless otherwise specified. ^2^ Female and male baseline characteristics were compared using the Mann–Whitney *U* test. Abbreviations: LDL, low-density lipoprotein cholesterol; HDL, high-density lipoprotein cholesterol; TC, total cholesterol; TAG, triglycerides; IL-6, interleukin-6; IL-1β, interleukin-1β; TNF-α, tumor necrosis factor-α; IL-10, interleukin-10.

**Table 3:** Baseline dietary intake per day and physical activity levels of study participants ^1^.

Variable ^2^	Total Population (*n* = 11)	Male (*n* = 5)	Female (*n* = 6)	*p*-Value ^3^
Energy, kJ	9169 (7230–10,448)	9169 (7413–10,503)	8903 (7212–10,502)	1.000
Protein, g % energy	95.8 (87.2–117.2) 20.2 (18.2–21.0)	100.4 (96.5–123.9) 21.0 (18.6–22.5)	90.0 (79.6–109.6) 19.9 (14.6–21.1)	0.100 0.361
Fat, g % energy	85.0 (76.2–116.8) 39.4 (37.0–40.9)	76.8 (68.3–112.8) 37.0 (31.3–39.7)	98.2 (77.1–127.3) 40.0 (39.3–45.2)	0.201 0.045
Saturated fat, g	31.2 (24.7–36.5)	24.7 (21.0–40.2)	32.6 (25.5–37.4)	0.584
MUFA, g	33.2 (29.8–52.4)	33.2 (27.6–48.1)	42.4 (31.6–56.8)	0.273
PUFA, g	14.7 (12.5–19.1)	14.2 (11.7–15.1)	17.3 (12.0–23.4)	0.361
Omega-3 fat, g	0.24 (0.13–0.75)	0.31 (0.18–0.74)	0.17 (0.12–1.17)	0.465
Omega-6 fat, g	12.3 (10.3–17.0)	11.4 (9.4–12.8)	15.2 (9.9–19.0)	0.201
Trans fat, g	1.17 (0.99–1.55)	1.05 (0.97–1.90)	1.24 (0.97–1.60)	0.855
Carbohydrate, g % energy	194.9 (147.2–225.9) 34.0 (30.6–38.2)	208.3 (148.2–241.5) 34.0 (31.1–41.5)	190.4 (143.3–230.4) 36.0 (30.0–38.7)	0.715 0.855
Sugar, g	73.3 (49.8–87.7)	73.3 (53.1–92.9)	73.7 (48.8–87.4)	0.584
Fibre, g	25.2 (21.7–33.9)	25.2 (23.1–33.0)	25.4 (19.5–36.2)	0.715
Sodium, mg	2268.7 (1937.7–3056.0)	2268.7 (1783.3–3048.7)	2170.3 (1721.9–3744.7)	1.000
Vegetable serves ^4^	3.80 (3.10–3.93)	3.71 (3.13–3.82)	3.91 (2.90–5.37)	0.273
Fruit serves ^4^	0.74 (0.37–1.20)	0.74 (0.15–2.81)	0.66 (0.38–0.99)	0.855
Grain serves ^4^ Wholegrain serves Refined grain serves	5.73 (5.18–6.97) 2.51 (0.95–4.06) 3.80 (2.56–6.00)	6.51 (5.44–7.88) 2.51 (1.34–3.38) 4.00 (2.14–6.43)	5.32 (5.06–7.78) 2.47 (0.84–4.66) 3.20 (2.20–5.06)	0.465 0.855 0.361
Total MET-min/week *n* (%) inactive (IPAQ 1) *n* (%) minimally active (IPAQ 2) *n* (%) HEPA active (IPAQ 3)	2079 (1152–4586) 1 (9) 5 (45) 5 (45)	2079 (807–5340) 1 (17) 2 (33) 3 (50)	3018 (1563–5025) 0 (0) 3 (50) 3 (50)	0.855 - - -

^1^ Values are median (Q_1_–Q_3_) unless otherwise indicated. ^2^ Average per day. ^3^ Female and male baseline characteristics were compared using the Mann–Whitney *U* test. ^4^ According to the Australian Guide to Healthy Eating. Abbreviations: MET, metabolic equivalent; MUFA, monounsaturated fatty acids; PUFA, polyunsaturated fatty acids; HEPA, health enhancing physical activity (a high active category).

**Table 4:** Postprandial plasma IL-6, IL-1β, TNF-α, and IL-10 measures for Anti-meal, Neutr-meal, and Pro-meal.

Outcome ^1^	Anti-Meal	Neutr-Meal	Pro-Meal	*p*-Value ^2^
IL-6 iAUC	70.8 (23.6–267.6)	59.2 (27.9–177.2)	78.8 (6.3–138.3)	0.695
IL-1β iAUC	9.6 (6.0–28.3)	13.4 (4.8–28.2)	6.9 (0.3–18.6)	0.695
TNF-α iAUC	114.6 (6.9–134.4)	66.9 (5.5–166.9)	72.7 (16.9–187.8)	0.761
IL-10 iAUC	177.0 (59.6–423.9)	201.6 (84.8–368.8)	149.3 (0–414.6)	0.761

^1^ Data are presented as median (IQR) (*n* = 11), and iAUC is represented in pg/mL·min. ^2^ Friedman test followed by pairwise comparisons using Wilcoxon signed-rank test were used to make comparisons between the three test meals. Abbreviations: Anti, anti-inflammatory; Neutr, neutral; Pro, proinflammatory; iAUC, incremental area under the curve.

**Table 5:** Postprandial plasma glucose, insulin, and serum lipid measures for Anti-meal, Neutr-meal and Pro-meal.

Outcome ^1^	Anti-Meal	Neutr-Meal	Pro-Meal	*p*-Value ^2^
Glucose iAUC	24.6 (8.9–124.0)	40.8 (24.1–67.2)	23.3 (9.8–43.2)	0.336
Insulin iAUC	1693.5 (734.8–3258.6)	2170.5 (1759.5–4014.0)	2767.5 (1597.7–2984.6)	0.336
TAG iAUC	27.0 (9.0–52.6) ^a^	63.7 (44.0–74.6) ^b^	135.6 (50.0–156.1) ^c^	0.009
TC iAUC	0 (0–0.64)	0 (0–0.44)	0 (0–0.50)	0.482

^1^ Data are presented as median (IQR) (*n* = 11), and iAUC is represented in mmol·min for glucose, TAG, and TC and in mIU/L.min for insulin. ^2^ Friedman test followed by pairwise comparisons using Wilcoxon signed-rank test were used to make comparisons between the three test meals. Unpaired letters abc indicates a significant difference between meals. Abbreviations: Anti, anti-inflammatory; Neutr, neutral; Pro, proinflammatory, TAG, triglycerides; TC, total cholesterol; iAUC, incremental area under the curve.

## Data Availability

The data presented in this study are available on request from the corresponding author. The data are not publicly available due to ethical obligations.

## Supplementary Materials

The following supporting information can be downloaded at: https://www.mdpi.com/article/10.3390/nu14071470/s1, Figure S1: Sequence of steps used to calculate the dietary inflammatory index (DII) for study meals; Figure S2: Postprandial plasma inflammatory markers; Table S1: Dietary inflammatory index calculation for the Pro-meal; Table S2: Dietary inflammatory index calculation for the Neutr-meal; Table S3: Dietary inflammatory index calculation for the Anti-meal; Table S4: Postprandial inflammatory and metabolic measures for the Anti-meal, Neutr-meal, and Pro-meal stratified by sex. References [29,58,59,60,61] are cited in the supplementary materials.
