# Supplementary material for: Meals That Differ in Nutrient Composition and Inflammatory Potential Do Not Differentially Impact Postprandial Circulating Cytokines in Older Adults above a Healthy Weight
Source: Nutrients. 2022 Apr 1;14(7):1470. doi: 10.3390/nu14071470 (PMC9003235; doi:10.3390/nu14071470)
Supplement: Supplementary file 1 [file nutrients-14-01470-s001.zip › nutrients-1633584-supplementary.pdf]

Supplementary Materials

Figures

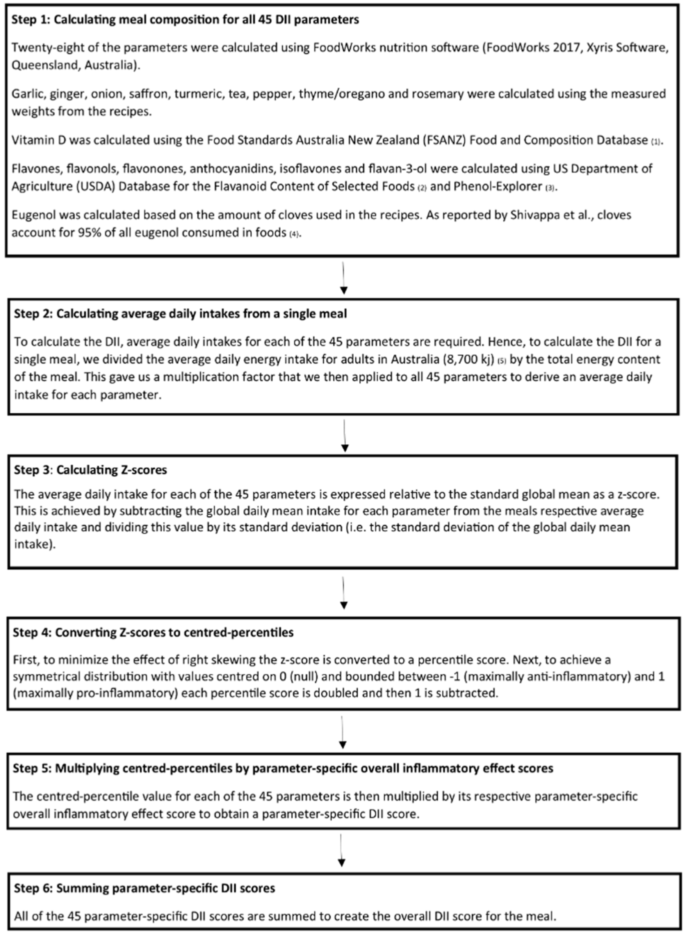

Figure S1. Sequence of steps used to calculate the Dietary Inflammatory Index (DII) for study meals. Adapted from Shivappa et al. (4).

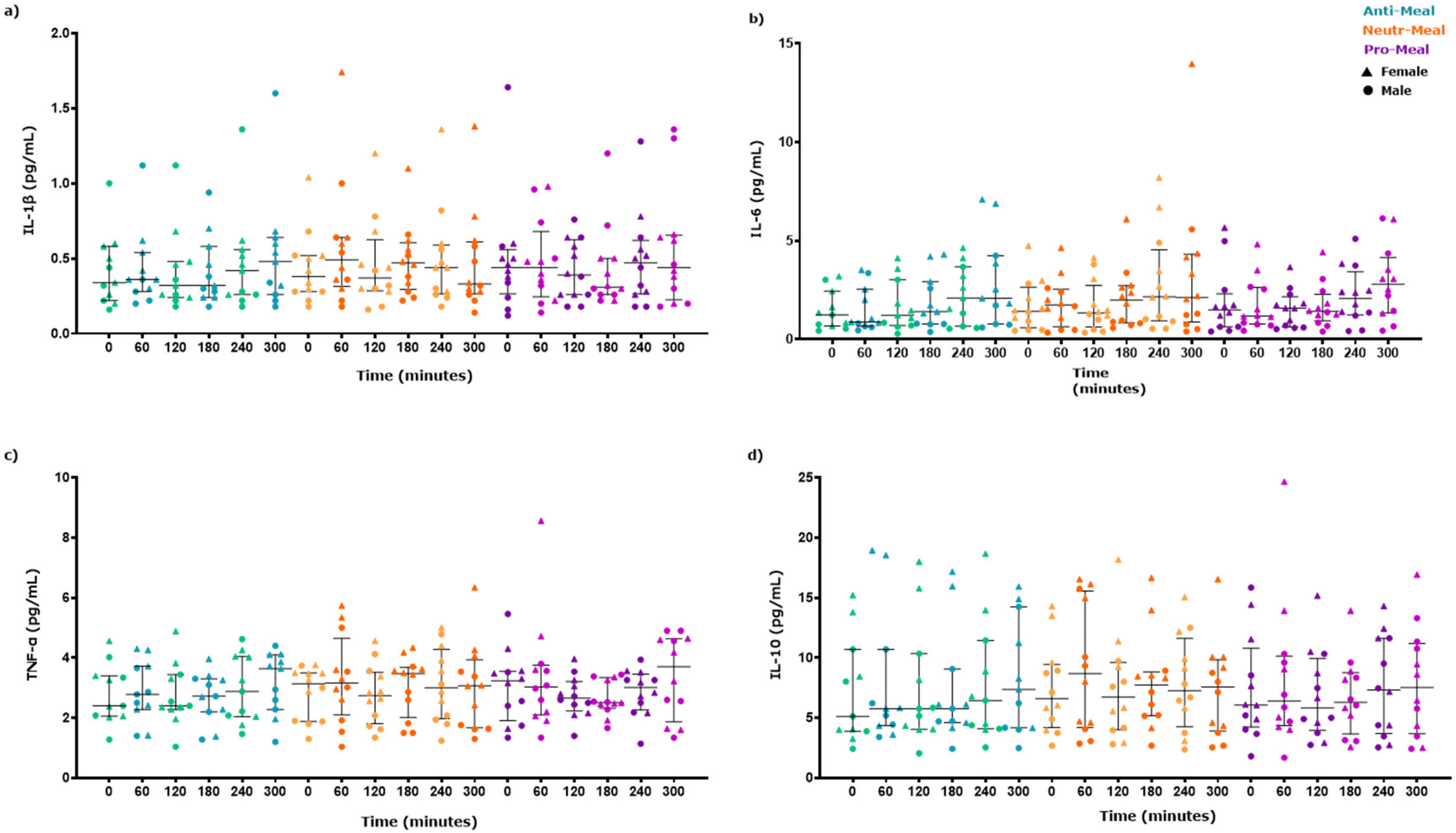

Figure S2. Post-prandial plasma (**a**) IL-1β, (**b**) IL-6, (**c**) TNF-α, and (**d**) IL-10 after ingestion of either the Anti-inflammatory meal, Neutral meal or Pro-inflammatory meal. Raw values are presented with median and interquartile range (IQR) for each time point. For all inflammatory markers one participant has been omitted for the Anti-inflammatory meal due to issues with cannulation. Abbreviations: Anti, anti-inflammatory; Neutr, neutral; Pro, pro-inflammatory.

Tables

Table S1. Dietary Inflammatory Index calculation for the Pro-inflammatory meal.

Food parameter	Global daily mean intake (units/d)[1]	Standard deviation[1]	Amount per serve (per meal)	Average daily intake[2]	Z-score[3]	Percentile score[4]	Centred-percentile[5]	Overall inflammatory effect score[6]	DII score[7]
**Alcohol (g)**	13.98	3.72	0.00	0.00	-3.76	0.00	-1.00	-0.28	0.28
**Vitamin B12 (μg)**	5.15	2.70	0.93	3.56	-0.59	0.28	-0.44	0.11	-0.05
**Vitamin B6 (mg)**	1.47	0.74	0.08	0.29	-1.59	0.06	-0.89	-0.37	0.32
**β-Carotene (μg)**	3718.00	1720.00	21.96	83.80	-2.11	0.02	-0.97	-0.58	0.56
**Caffeine (g)**	8.05	6.67	0.00	0.00	-1.21	0.11	-0.77	-0.11	0.08
**Carbohydrate (g)**	272.20	40.00	25.22	96.25	-4.40	0.00	-1.00	0.10	-0.10
**Cholesterol (mg)**	279.40	51.20	75.02	286.31	0.13	0.55	0.11	0.11	0.01
**Energy (kJ)**	8602.30	1414.19	2279.67	8700.00	0.07	0.53	0.06	0.18	0.01
**Eugenol (mg)**	0.01	0.08	0.00	0.00	-0.13	0.45	-0.10	-0.14	0.01
**Total fat (g)**	71.40	19.40	39.33	150.10	4.06	1.00	1.00	0.30	0.30
**Fibre (g)**	18.80	4.90	1.96	7.48	-2.31	0.01	-0.98	-0.66	0.65
**Folic acid (μg)**	273.00	70.70	55.95	213.51	-0.84	0.20	-0.60	-0.19	0.11
**Garlic (g)**	4.35	2.90	0.00	0.00	-1.50	0.07	-0.87	-0.41	0.36
**Ginger (g)**	59.00	63.20	0.00	0.00	-0.93	0.18	-0.65	-0.45	0.29
**Iron (mg)**	13.35	3.71	0.90	3.44	-2.67	0.00	-0.99	0.03	-0.03

Food parameter	Global daily mean intake (units/d)[1]	Standard deviation[1]	Amount per serve (per meal)	Average daily intake[2]	Z-score[3]	Percentile score[4]	Centred-percentile[5]	Overall inflammatory effect score[6]	DII score[7]
**Magnesium (mg)**	310.10	139.40	30.53	116.52	-1.39	0.08	-0.84	-0.48	0.40
**MUFA (g)**	27.00	6.10	13.69	52.25	4.14	1.00	1.00	-0.01	-0.01
**Niacin (mg)**	25.90	11.77	7.29	27.82	0.16	0.56	0.13	-0.25	-0.03
**n-3 Fatty acids (g)**	1.06	1.06	0.34	1.31	0.23	0.59	0.19	-0.44	-0.08
**n-6 Fatty acids (g)**	10.80	7.50	1.94	7.40	-0.45	0.32	-0.35	-0.16	0.06
**Onion (g)**	35.90	18.40	0.00	0.00	-1.95	0.03	-0.95	-0.30	0.29
**Protein (g)**	79.40	13.90	22.21	84.75	0.38	0.65	0.30	0.02	0.01
**PUFA (g)**	13.88	3.76	2.50	9.53	-1.16	0.12	-0.75	-0.34	0.25
**Riboflavin (mg)**	1.70	0.79	0.21	0.79	-1.16	0.12	-0.75	-0.07	0.05
**Saffron (g)**	0.37	1.78	0.00	0.00	-0.21	0.42	-0.16	-0.14	0.02
**Saturated fat (g)**	28.60	8.00	20.45	78.04	6.18	1.00	1.00	0.37	0.37
**Selenium (μg)**	67.00	25.10	18.65	71.16	0.17	0.57	0.13	-0.19	-0.03
**Thiamine (mg)**	1.70	0.66	0.22	0.84	-1.30	0.10	-0.81	-0.10	0.08
**Trans fat (g)**	3.15	3.75	1.00	3.83	0.18	0.57	0.14	0.23	0.03
**Turmeric (mg)**	533.60	754.30	0.00	0.00	-0.71	0.24	-0.52	-0.79	0.41
**Vitamin A (RE)**	983.90	518.60	53.84	205.48	-1.50	0.07	-0.87	-0.40	0.35
**Vitamin C (mg)**	118.20	43.46	0.25	0.95	-2.70	0.00	-0.99	-0.42	0.42
**Vitamin D**	6.26	2.21	0.03	0.10	-2.79	0.00	-0.99	-0.45	0.44

Food parameter	Global daily mean intake (units/d)[1]	Standard deviation[1]	Amount per serve (per meal)	Average daily intake[2]	Z-score[3]	Percentile score[4]	Centred-percentile[5]	Overall inflammatory effect score[6]	DII score[7]
**(μg)**									
**Vitamin E (mg)**	8.73	1.49	0.64	2.44	-4.22	0.00	-1.00	-0.42	0.42
**Zinc (mg)**	9.84	2.19	1.88	7.16	-1.22	0.11	-0.78	-0.31	0.24
**Green and black tea (g)**	1.69	1.53	0.00	0.00	-1.10	0.13	-0.73	-0.54	0.39
**Flavan-3-ol (mg)**	95.80	85.90	0.00	0.00	-1.12	0.13	-0.74	-0.42	0.31
**Flavones (mg)**	1.55	0.07	0.00	0.00	-22.14	0.00	-1.00	-0.62	0.62
**Flavonols (mg)**	17.70	6.79	0.00	0.00	-2.61	0.00	-0.99	-0.47	0.46
**Flavonones (mg)**	11.70	3.82	0.00	0.00	-3.06	0.00	-1.00	-0.25	0.25
**Anthocyanidin (mg)**	18.05	21.14	0.00	0.00	-0.85	0.20	-0.61	-0.13	0.08
**Isoflavones (mg)**	1.20	0.20	0.00	0.00	-6.00	0.00	-1.00	-0.59	0.59
**Pepper (g)**	10.00	7.07	0.00	0.00	-1.41	0.08	-0.84	-0.13	0.11
**Thyme and oregano (mg)**	0.33	0.99	0.00	0.00	-0.33	0.37	-0.26	-0.10	0.03
**Rosemary (mg)**	1.00	15.00	0.00	0.00	-0.07	0.47	-0.05	-0.01	0.00
**Overall DII Score[8]**								**+9.36**	

[1]As calculated and described by Shivappa et al. (4). [2]To calculate a DII score for a single meal, we first divided the average daily energy intake for adults in Australia (8,700 kJ) (5) by the total energy content of the meal. This gave us a multiplication factor (3.726) that we then applied to all 45 parameters to derive an 'average daily intake' for each parameter. [3]Z-scores were derived by subtracting the global daily mean intake for each parameter from their respective average daily intake and dividing this value by its standard deviation. [4]To minimize the effect of right skewing the z-score was converted to a percentile score. [5]To achieve a symmetrical distribution (i.e. centred-percentile) with values centred on 0 (null) and bounded between -1 (maximally anti-inflammatory) and 1 (maximally pro-inflammatory), each percentile score was doubled and then 1 was subtracted. [6]As calculated and described by Shivappa et al. (4). [7]The centred-percentile for each parameter was multiplied by its respective overall inflammatory effect score to obtain a DII score for each parameter. [8]All of the 45 parameter-specific DII scores were summed to create the overall DII score for the meal. Abbreviations: DII, Dietary Inflammatory Index; MUFA, monounsaturated fatty acids; PUFA, polyunsaturated fatty acids.

Table S2. Dietary Inflammatory Index calculation for the Neutral meal.

Food parameter	Global daily mean intake (units/d)[1]	Standard deviation[1]	Amount per serve (per meal)	Average daily intake[2]	Z-score[3]	Percentile score[4]	Centred-percentile[5]	Overall inflammatory effect score[6]	DII score[7]
**Alcohol (g)**	13.98	3.72	0.00	0.00	-3.76	0.00	-1.00	-0.28	0.28
**Vitamin B12 (μg)**	5.15	2.70	1.00	4.05	-0.41	0.34	-0.32	0.11	-0.03
**Vitamin B6 (mg)**	1.47	0.74	0.61	2.45	1.32	0.91	0.81	-0.37	-0.30
**β-Carotene (μg)**	3718.00	1720.00	2720.90	11011.34	4.24	1.00	1.00	-0.58	-0.58
**Caffeine (g)**	8.05	6.67	0.00	0.00	-1.21	0.11	-0.77	-0.11	0.08
**Carbohydrate (g)**	272.20	40.00	42.82	173.27	-2.47	0.01	-0.99	0.10	-0.10
**Cholesterol (mg)**	279.40	51.20	71.00	287.33	0.15	0.56	0.12	0.11	0.01
**Energy (kJ)**	8602.30	1414.19	2149.77	8700.00	0.07	0.53	0.06	0.18	0.01
**Eugenol (mg)**	0.01	0.08	0.00	0.00	-0.13	0.45	-0.10	-0.14	0.01
**Total fat (g)**	71.40	19.40	246.00	995.55	47.64	1.00	1.00	0.30	0.30
**Fibre (g)**	18.80	4.90	15.47	62.60	8.94	1.00	1.00	-0.66	-0.66
**Folic acid (μg)**	273.00	70.70	101.29	409.92	1.94	0.97	0.95	-0.19	-0.18
**Garlic (g)**	4.35	2.90	1.25	5.06	0.24	0.60	0.19	-0.41	-0.08
**Ginger (g)**	59.00	63.20	0.00	0.00	-0.93	0.18	-0.65	-0.45	0.29
**Iron (mg)**	13.35	3.71	5.09	20.61	1.96	0.97	0.95	0.03	0.03
**Magnesium (mg)**	310.10	139.40	121.16	490.33	1.29	0.90	0.80	-0.48	-0.39

Food parameter	Global daily mean intake (units/d)[1]	Standard deviation[1]	Amount per serve (per meal)	Average daily intake[2]	Z-score[3]	Percentile score[4]	Centred-percentile[5]	Overall inflammatory effect score[6]	DII score[7]
MUFA (g)	27.00	6.10	5.32	21.53	-0.90	0.19	-0.63	-0.01	0.01
Niacin (mg)	25.90	11.77	20.45	82.78	4.83	1.00	1.00	-0.25	-0.25
n-3 Fatty acids (g)	1.06	1.06	0.36	1.47	0.39	0.65	0.30	-0.44	-0.13
n-6 Fatty acids (g)	10.80	7.50	1.05	4.27	-0.87	0.19	-0.62	-0.16	0.10
Onion (g)	35.90	18.40	28.75	116.35	4.37	1.00	1.00	-0.30	-0.30
Protein (g)	79.40	13.90	43.12	174.49	6.84	1.00	1.00	0.02	0.02
PUFA (g)	13.88	3.76	1.46	5.90	-2.12	0.02	-0.97	-0.34	0.33
Riboflavin (mg)	1.70	0.79	0.38	1.53	-0.22	0.41	-0.17	-0.07	0.01
Saffron (g)	0.37	1.78	0.00	0.00	-0.21	0.42	-0.16	-0.14	0.02
Saturated fat (g)	28.60	8.00	6.21	25.15	-0.43	0.33	-0.33	0.37	-0.12
Selenium (µg)	67.00	25.10	24.14	97.71	1.22	0.89	0.78	-0.19	-0.15
Thiamine (mg)	1.70	0.66	0.37	1.50	-0.30	0.38	-0.24	-0.10	0.02
Trans fat (g)	3.15	3.75	0.25	1.00	-0.57	0.28	-0.43	0.23	-0.10
Turmeric (mg)	533.60	754.30	0.00	0.00	-0.71	0.24	-0.52	-0.79	0.41
Vitamin A (RE)	983.90	518.60	513.49	2078.07	2.11	0.98	0.97	-0.40	-0.39
Vitamin C (mg)	118.20	43.46	88.53	358.28	5.52	1.00	1.00	-0.42	-0.42
Vitamin D (µg)	6.26	2.21	6.81	27.33	9.53	1.00	1.00	-0.45	-0.45
Vitamin E (mg)	8.73	1.49	4.02	16.26	5.06	1.00	1.00	-0.42	-0.42

Food parameter	Global daily mean intake (units/d)[1]	Standard deviation[1]	Amount per serve (per meal)	Average daily intake[2]	Z-score[3]	Percentile score[4]	Centred-percentile[5]	Overall inflammatory effect score[6]	DII score[7]
**Zinc (mg)**	9.84	2.19	3.78	15.28	2.48	0.99	0.99	-0.31	-0.31
**Green and black tea (g)**	1.69	1.53	0.00	0.00	-1.10	0.13	-0.73	-0.54	0.39
**Flavan-3-ol (mg)**	95.80	85.90	2.01	8.06	-1.02	0.15	-0.69	-0.42	0.29
**Flavones (mg)**	1.55	0.07	0.55	2.23	9.65	1.00	1.00	-0.62	-0.62
**Flavonols (mg)**	17.70	6.79	13.78	55.28	5.53	1.00	1.00	-0.47	-0.47
**Flavonones (mg)**	11.70	3.82	0.00	0.00	-3.06	0.00	-1.00	-0.25	0.25
**Anthocyanidin (mg)**	18.05	21.14	0.00	0.00	-0.85	0.20	-0.61	-0.13	0.08
**Isoflavones (mg)**	1.20	0.20	0.00	0.00	-5.98	0.00	-1.00	-0.59	0.59
**Pepper (g)**	10.00	7.07	0.00	0.00	-1.41	0.08	-0.84	-0.13	0.11
**Thyme and oregano (mg)**	0.33	0.99	0.00	0.00	-0.33	0.37	-0.26	-0.10	0.03
**Rosemary (mg)**	1.00	15.00	0.00	0.00	-0.07	0.47	-0.05	-0.01	0.00
**Overall DII Score[8]**								**-2.76**	

[1]As calculated and described by Shivappa et al. (4). [2]To calculate a DII score for a single meal, we first divided the average daily energy intake for adults in Australia (8,700 kJ) (5) by the total energy content of the meal. This gave us a multiplication factor (3.726) that we then applied to all 45 parameters to derive an 'average daily intake' for each parameter. [3]Z-scores were derived by subtracting the global daily mean intake for each parameter from their respective average daily intake and dividing this value by its standard deviation. [4]To minimize the effect of right skewing the z-score was converted to a percentile score. [5]To achieve a symmetrical distribution (i.e. centred-percentile) with values centred on 0 (null) and bounded between -1 (maximally anti-inflammatory) and 1 (maximally pro-inflammatory), each percentile score was doubled and then 1 was subtracted. [6]As calculated and described by Shivappa et al. (4). [7]The centred-percentile for each parameter was multiplied by its respective overall inflammatory effect score to obtain a DII score for each parameter. [8]All of the 45 parameter-specific DII scores were summed to create the overall DII score for the meal. Abbreviations: DII, Dietary Inflammatory Index; MUFA, monounsaturated fatty acids; PUFA, polyunsaturated fatty acids.

Table S3. Dietary Inflammatory Index calculation for the Anti-inflammatory meal.

Food parameter	Global daily mean intake (units/d)[1]	Standard deviation[1]	Amount per serve (per meal)	Average daily intake[2]	Z-score[3]	Percentile score[4]	Centred-percentile[5]	Overall inflammatory effect score[6]	DII score[7]
**Alcohol (g)**	13.98	3.72	0.00	0.00	-3.76	0.00	-1.00	-0.28	0.28
**Vitamin B12 (μg)**	5.15	2.70	0.30	1.12	-1.49	0.07	-0.86	0.11	-0.09
**Vitamin B6 (mg)**	1.47	0.74	1.22	4.54	4.15	1.00	1.00	-0.37	-0.36
**β-Carotene (μg)**	3718.00	1720.00	2699.62	10058.21	3.69	1.00	1.00	-0.58	-0.58
**Caffeine (g)**	8.05	6.67	0.00	0.00	-1.21	0.11	-0.77	-0.11	0.08
**Carbohydrate (g)**	272.20	40.00	53.15	198.02	-1.85	0.03	-0.94	0.10	-0.09
**Cholesterol (mg)**	279.40	51.20	22.03	82.09	-3.85	0.00	-1.00	0.11	-0.11
**Energy (kJ)**	8602.30	1414.19	2335.08	8700.00	0.07	0.53	0.06	0.18	0.01
**Eugenol (mg)**	0.01	0.08	22.04	82.11	1026.22	1.00	1.00	-0.14	-0.14
**Total fat (g)**	71.40	19.40	21.89	81.57	0.52	0.70	0.40	0.30	0.12
**Fibre (g)**	18.80	4.90	19.01	70.81	10.61	1.00	1.00	-0.66	-0.66
**Folic acid (μg)**	273.00	70.70	117.26	436.87	2.32	0.99	0.98	-0.19	-0.19
**Garlic (g)**	4.35	2.90	2.00	7.45	1.07	0.86	0.72	-0.41	-0.29
**Ginger (g)**	59.00	63.20	0.88	3.26	-0.88	0.19	-0.62	-0.45	0.28
**Iron (mg)**	13.35	3.71	7.24	26.96	3.67	1.00	1.00	0.03	0.03
**Magnesium (mg)**	310.10	139.40	214.65	799.73	3.51	1.00	1.00	-0.48	-0.48
**MUFA (g)**	27.00	6.10	9.36	34.88	1.29	0.90	0.80	-0.01	-0.01

Food parameter	Global daily mean intake (units/d)[1]	Standard deviation[1]	Amount per serve (per meal)	Average daily intake[2]	Z-score[3]	Percentile score[4]	Centred-percentile[5]	Overall inflammatory effect score[6]	DII score[7]
**Niacin (mg)**	25.90	11.77	13.20	49.18	1.98	0.98	0.95	-0.25	-0.23
**n-3 Fatty acids (g)**	1.06	1.06	4.62	17.21	15.23	1.00	1.00	-0.44	-0.44
**n-6 Fatty acids (g)**	10.80	7.50	2.53	9.42	-0.18	0.43	-0.15	-0.16	0.02
**Onion (g)**	35.90	18.40	19.25	71.72	1.95	0.97	0.95	-0.30	-0.29
**Protein (g)**	79.40	13.90	25.72	95.82	1.18	0.88	0.76	0.02	0.02
**PUFA (g)**	13.88	3.76	7.18	26.74	3.42	1.00	1.00	-0.34	-0.34
**Riboflavin (mg)**	1.70	0.79	0.46	1.70	-0.01	0.50	0.00	-0.07	0.00
**Saffron (g)**	0.37	1.78	0.03	0.11	-0.15	0.44	-0.12	-0.14	0.02
**Saturated fat (g)**	28.60	8.00	3.42	12.75	-1.98	0.02	-0.95	0.37	-0.36
**Selenium (μg)**	67.00	25.10	15.95	59.41	-0.30	0.38	-0.24	-0.19	0.05
**Thiamine (mg)**	1.70	0.66	0.65	2.41	1.10	0.86	0.73	-0.10	-0.07
**Trans fat (g)**	3.15	3.75	0.02	0.08	-0.82	0.21	-0.59	0.23	-0.13
**Turmeric (mg)**	533.60	754.30	875.00	3260.06	3.61	1.00	1.00	-0.79	-0.78
**Vitamin A (RE)**	983.90	518.60	456.00	1698.96	1.57	0.94	0.88	-0.40	-0.35
**Vitamin C (mg)**	118.20	43.46	74.09	276.03	3.63	1.00	1.00	-0.42	-0.42
**Vitamin D (μg)**	6.26	2.21	1.5	5.59	-0.30	0.38	-0.24	-0.45	0.11
**Vitamin E (mg)**	8.73	1.49	7.97	29.69	14.07	1.00	1.00	-0.42	-0.42
**Zinc**	9.84	2.19	3.69	13.75	1.06	0.86	0.71	-0.31	-0.22

Food parameter	Global daily mean intake (units/d)[1]	Standard deviation[1]	Amount per serve (per meal)	Average daily intake[2]	Z-score[3]	Percentile score[4]	Centred-percentile[5]	Overall inflammatory effect score[6]	DII score[7]
**(mg)**									
**Green and black tea (g)**	1.69	1.53	0.00	0.00	-1.10	0.13	-0.73	-0.54	0.39
**Flavan-3-ol (mg)**	95.80	85.90	2.09	7.77	-1.02	0.15	-0.69	-0.42	0.29
**Flavones (mg)**	1.55	0.07	18.57	69.18	966.12	1.00	1.00	-0.62	-0.62
**Flavonols (mg)**	17.70	6.79	15.49	57.73	5.90	1.00	1.00	-0.47	-0.47
**Flavonones (mg)**	11.70	3.82	100.06	372.80	94.53	1.00	1.00	-0.25	-0.25
**Anthocyanidin (mg)**	18.05	21.14	28.28	105.36	4.13	1.00	1.00	-0.13	-0.13
**Isoflavones (mg)**	1.20	0.20	0.02	0.06	-5.68	0.00	-1.00	-0.59	0.59
**Pepper (g)**	10.00	7.07	0.28	1.02	-32.64	0.00	-1.00	-0.13	0.13
**Thyme and oregano (mg)**	0.33	0.99	875.00	3260.06	3.73	1.00	1.00	-0.10	-0.10
**Rosemary (mg)**	1.00	15.00	875.00	3428.25	3.92	1.00	1.00	-0.01	-0.01
**Overall DII Score[8]**								**-6.24**	

[1]*As calculated and described by Shivappa et al. (4).* [2]*To calculate a DII score for a single meal, we first divided the average daily energy intake for adults in Australia (8,700 kJ) (5) by the total energy content of the meal. This gave us a multiplication factor (3.726) that we then applied to all 45 parameters to derive an 'average daily intake' for each parameter.* [3]*Z-scores were derived by subtracting the global daily mean intake for each parameter from their respective average daily intake and dividing this value by its standard deviation.* [4]*To minimize the effect of right skewing the z-score was converted to a percentile score.* [5]*To achieve a symmetrical distribution (i.e. centred-percentile) with values centred on 0 (null) and bounded between -1 (maximally anti-inflammatory) and 1 (maximally pro-inflammatory), each percentile score was doubled and then 1 was subtracted.* [6]*As calculated and described by Shivappa et al. (4).* [7]*The centred-percentile for each parameter was multiplied by its respective overall inflammatory effect score to obtain a DII score for each parameter.* [8]*All of the 45 parameter-specific DII scores were summed to create the overall DII score for the meal. Abbreviations: DII, Dietary Inflammatory Index; MUFA, monounsaturated fatty acids; PUFA, polyunsaturated fatty acids.*

Table S4. Postprandial inflammatory and metabolic measures for the Anti-meal, Neutr-meal and Pro-meal stratified by sex.

Outcome[1]	sex	n	Anti-meal	Neutr-meal	Pro-meal	P-value[2]
IL-6 iAUC (pg/mL·min)	Female	6	236.7 (52.7 – 476.7)	157.6 (85.8 – 416.8)	120.8 (39.4 – 166.9)	0.311
	Male	5	36.0 (5.1 – 79.7)	58.2 (18.7 – 58.8)	24.6 (3.2 – 142.9)	0.549
IL-1β iAUC (pg/mL·min)	Female	6	24.0 (4.5 – 28.5)	20.8 (4.0 – 44.6)	3.4 (0.2 – 19.9)	0.311
	Male	5	7.7 (3.3 – 30.4)	12.9 (4.0 – 22.9)	15.7 (3.5 – 20.4)	0.549
TNF-α iAUC (pg/mL·min)	Female	6	130.6 (5.1 – 146.7)	121.5 (0.3 – 288.5)	67.2 (12.8 – 174.1)	0.607
	Male	5	72.0 (7.0 – 123.2)	64.2 (6.0 – 116.9)	72.7 (9.6 – 190.2)	0.165
IL10 iAUC (pg/mL·min)	Female	6	355.0 (102.6 – 792.6)	301.8 (104.1 – 431.4)	130.8 (0 – 484.6)	0.513
	Male	5	135.3 (29.8 – 216.0)	102.6 (51.4 – 301.6)	163.6 (24.3 – 430.2)	0.819
Glucose iAUC (mmol/L·min)	Female	6	24.2 (8.2 – 170.6)	34.0 (21.3 – 75.9)	14.9 (5.1 – 49.0)	0.135
	Male	5	34.0 (4.5- 88.3)	46.3 (23.3 – 63.8)	36.8 (19.2 – 61.8)	1.000
Insulin iAUC (mIU/L·min)	Female	6	1752.0 (1106.4 – 3391.6)	2152.5 (1538.1 – 3629.7)	2932.5 (1500.4 – 3427.6)	0.846
	Male	5	1693.5 (711.6 – 2775.0)	3259.5 (1707.8 – 4531.5)	2440.5 (1303.5 – 3840.8)	0.074
TAG iAUC (mmol/L·min)	Female	6	20.1 (8.8 - 47.0)[a]	58.8 (30.2 - 77.8)[a]	121.9 (43.3 - 230.5)[b]	0.042
	Male	5	31.9 (6.0 - 61.1)	65.1 (53.9 - 76.1)	140.0 (47.6 - 152.5)	0.165
TC iAUC (mmol/L·min)	Female	6	0.32 (0 – 17.52)	0 (0 – 4.42)	0 (0 – 1.77)	0.931
	Male	5	0 (0 – 0)	0.01 (0 – 9.39)	0 (0 – 0.52)	0.174

[1]*Data are presented as median* ($Q_1 – Q_3$)*, inflammatory marker, glucose and insulin are analysed from blood plasma and triglycerides and total cholesterol are analysed from blood serum.*

[2] *Friedman test followed by pairwise comparisons using Wilcoxon signed-rank test were used to make comparisons between the three test meals. *Unpaired letters [ab] indicates a significant difference between meals. Abbreviations: A, anti-inflammatory; N, neutral; P, pro-inflammatory, TAG, triglycerides; TC, total cholesterol; iAUC, incremental area under curve.*

References

1. Food Standards Australia New Zealand. Australian Food Composition Database. 2019. Available online: https://www.foodstandards.gov.au/science/monitoringnutrients/afcd/Pages/default.aspx (accessed on 20 February 2020).
2. U.S. Department of Agriculture. USDA Database for the Flavonoid Content of Selected Foods, Maryland. 2016. Available online: https://data.nal.usda.gov/dataset/usda-database-flavonoid-content-selected-foods-release-32- november-2015 (accessed on 20 February 2020).
3. Rothwell, J.A.; Perez-Jimenez, J.; Neveu, V.; Medina-Remon, A.; M'hiri, N.; García-Lobato, P.; Manach, C.; Knox, C.; Eisner, R.; Wishart, D.S.; et al. Phenol-Explorer 3.0: A major update of the Phenol-Explorer database to incorporate data on the effects of food processing on polyphenol content. *Database* **2013**, *2013*, bat070. https://doi.org/10.1093/database/bat070.
4. Shivappa, N.; Steck, S.E.; Hurley, T.G.; Hussey, J.R.; Hébert, J.R. Designing and developing a literature derived, population-based dietary inflammatory index. *Public Health Nutr.* **2014**, *17*, 1689–1696.
5. Australian Bureau of Statistics. 4802.0—National Nutrition Survey: Selected Highlights, Australia, 1995. 1997. Available online: https://www.abs.gov.au/AUSSTATS/abs@.nsf/DetailsPage/4802.01995?OpenDocument (accessed on 20 February 2020).
